# Isolation of *Rickettsia rickettsii* in Rocky Mountain Spotted Fever Outbreak, Panama

**DOI:** 10.3201/eid2704.201606

**Published:** 2021-04

**Authors:** Yamitzel Zaldívar, Michelle Hernández, Lillian Domínguez, Lisseth Saénz, Santiago Montilla, Maria E. Barnett de Antinori, Felipe S. Krawczak, Sergio Bermúdez

**Affiliations:** Gorgas Memorial Institute for Health Research, Panama City, Panama (Y. Zaldívar, M. Hernández, L. Domínguez, L. Saénz, S. Montilla, M.E.B. de Antinori, S. Bermúdez);; School of Veterinary Medicine and Animal Science, Federal University of Goiás–UFG, Goiânia, Brazil (F.S. Krawczak);; Coiba Scientific Station, Panama City (S. Bermúdez)

**Keywords:** *Rickettsia rickettsii* spotted fever group, Rocky Mountain spotted fever, human cases, isolation, bacteria, Panama, rickettsiosis, rickettsia, vector-borne infections

## Abstract

We report new cases of Rocky Mountain spotted fever in patients from Kinkantu, Ngäbe-Bugle indigenous comarca, Panama. We isolated *Rickettsia rickettsii* in cell culture after intraperitoneal inoculation of guinea pigs with tissues from a deceased patient. Our results indicate that Rocky Mountain spotted fever is emerging in this region.

Rocky Mountain spotted fever (RMSF) causes severe cases of rickettsiosis and is considered a principal tickborne pathogen in the Americas (*1*). Clinical suspicion is crucial for timely therapy with doxycycline to prevent severe illness and death (*1*). In Panama, 5 cases of RMSF were reported during 1950–1953, of which 2 were fatal; since 2004, a total of 19 new cases have been reported in Panama, with 13 fatal cases (*2*). We report new cases of RMSF from Piedra Roja, a rural village of Kankintu, Ngäbe-Bugle indigenous comarca, located at 750 m above sea level in the western mountainous region of Panama without road access.

In February 2019, a total of 7 persons 3–20 years of age from a family cluster had a clinical picture characterized by temperatures of 39°C–41°C (100%), generalized exanthema (100%), diarrhea and vomiting (86%), headaches (71%), severe dehydration (57%), abdominal pain (43%), and hepatomegaly and jaundice (29%). The patients reported no history of recent tick bites or attachment; according to each patient, the duration of symptoms varied from 9 to 11 days. Of these 7 patients, 2 recovered after treatment with doxycycline, 1 recovered without treatment with doxycycline, and 4 died. 

We diagnosed rickettsiosis by PCR on blood and samples of spleen, liver, brain and lung, using the Rr190.70p and Rr190.602n primers, which amplify a ≈532 bp fragment of outer membrane protein gene (*ompA*) (*3*). Samples of blood, liver, and spleen from 6 patients yielded *ompA* amplicons, of which 3 generated DNA sequences 100% identical to *R. rickettsii* were deposited in GenBank (accession nos. MF678551.1, KX363464.1, and CP006010.1).

Tissue samples were recovered during the autopsy of 1 patient and stored at −40°C. Because this temperature is higher than that recommended to keep *Rickettsia* viable, we inoculated 1 guinea pig (*Cavia porcellus*) with tissue homogenate to avoid rickettsial load loss at the moment of isolation. These animals have been reported as amplifier hosts for *R. rickettsii* (*4*,*5*). Therefore, we inoculated a homogenate of spleen, liver, and lung tissues into an adult male guinea pig before starting the isolation through cell culture. The animal did not have a fever (rectal temperature ≤39.6°C) but died on the 7th day postinoculation (dpi). We extracted and macerated the liver, spleen, brain, and lungs to inoculate 5 additional guinea pigs (second passage), following Krawczak et al. (*4*). Of these, 2 animals died <24 hours later and were eliminated from the study, 1 developed high fever (>40.0°C) at 4 dpi that persisted until 6 dpi, and 2 remained afebrile but died at 4–5 dpi. We isolated rickettsiae in cell culture from a febrile (>39.6°C) guinea pig that was euthanized at 6 dpi. We inoculated fragments of liver, spleen, and lungs into flasks containing a monolayer of Vero cells, as previously described (*5**,**6*). We considered a rickettsial isolate to be established in the laboratory after third passages, each reaching an infected cell level >90% (*6**,**7*). We successfully isolated rickettsiae in Vero cells of homogenate derived from a 3-guinea-pig passage.

We extracted DNA from infected cells following Krawczak et al. (*4*) using a PCR targeting *gltA* (401 bp), *ompA* (532 bp), and *ompB* (511 bp) (*3**,**6*). Sequenced PCR products showed a 100% identity with *R. rickettsii gltA* (GenBank accession nos. CP018914.1, CP018913.1, CP006010.1, CP006009.1, and CP000766.3), *ompA* (GenBank accession nos. MF678551.1 and MF988095.1), and *ompB* (GenBank accession nos. CP018914.1, CP018913.1, CP006010.1, CP006009.1, and CP000766.3). We deposited DNA of an isolate in GenBank (accession no. MT814706 for the *gltA* gene, MT268770 for the *ompA* gene, and MT814707 for the *ompB* gene). We designated the *R. rickettsii* isolate as strain NB, for Ngäbe Bugle, and deposited it in the Gorgas Memorial Institute at Biosafety Level 3.

The diagnosis of severe cases of RMSF in Piedra Roja represents a new locality for this disease in Panama. RMSF has been reported previously from the provinces of Panama, Panama Oeste, and Colon, associated with the distribution of *Amblyomma mixtum* and *Rhipicephalus sanguineus* s.l. ticks (*2**,**8*). More studies will be needed to determine the ecology related to these cases.

We were able to isolate *R. rickettsii* from infected tissues stored at −40°C, which is higher than the recommended temperature of −80°C for preserving tissues (*9*). Because of the relevance of *R. rickettsii* as a pathogen, the isolation of strains favors obtaining antigens for serologic tests and for further studies to determine the genetic and pathogenic differences between strains. Currently, >30 genotypes of *R. rickettsii* exist, with different degrees of pathogenicity; therefore, a more representative sample of isolates may make it possible to estimate variations among different populations (*10*).

In summary, we investigated an outbreak of RMSF in Piedra Roja, a rural village in western Panama, an area where this disease had not previously been reported. Clinicians should remain aware of the possibility of *R. rickettsii* infection in this region.

## References

[1] Oliveira SV, Caldas EP, Colombo S, Gazeta GS, Labruna MB, Santos FC, et al. A fatal case of Brazilian spotted fever in a non-endemic area in Brazil: the importance of having health professionals who understand the disease and its areas of transmission. Rev Soc Bras Med Trop. 2016;49:653–5. 10.1590/0037-8682-0088-2016 27812666

[2] Bermúdez S, Domínguez L, Suárez J, Daza C, Cumbrera A, González J. Past and present of rickettsiosis in Panamá [in Spanish]. Panama City: Instituto Conmemorativo Gorgas de Estudios de la Salud, Panamá; 2018.

[3] Regnery RL, Spruill CL, Plikaytis BD. Genotypic identification of rickettsiae and estimation of intraspecies sequence divergence for portions of two rickettsial genes. J Bacteriol. 1991;173:1576–89. 10.1128/JB.173.5.1576-1589.1991 1671856 PMC207306

[4] Krawczak FS, Nieri-Bastos FA, Nunes FP, Soares JF, Moraes-Filho J, Labruna MB. Rickettsial infection in *Amblyomma cajennense* ticks and capybaras (*Hydrochoerus hydrochaeris*) in a Brazilian spotted fever-endemic area. Parasit Vectors. 2014;7:7. 10.1186/1756-3305-7-7 24387674 PMC3892071

[5] Stokes J, Walker D, Varela-Stokes A. The guinea pig model for tick-borne spotted fever rickettsioses: a second look. Ticks Tick-borne Dis. 2020;11:101538. 10.1016/j.ttbdis.2020.101538 32993947 PMC7530330

[6] Paddock, C.D., Allerdice, M., Karpathy, S.E., Nicholson, W.L., Levin, M.L., Smith, T.C. et al. Unique strain of *Rickettsia parkeri* associated with the hard tick *Dermacentor parumapertus* Neumann in the western United States. Appl Environ Microbiol. 2017;83:e03463-16. 10.1128/AEM.03463-16 28213544 PMC5394329

[7] Labruna MB, Santos FC, Ogrzewalska M, Nascimento EM, Colombo S, Marcili A, et al. Genetic identification of rickettsial isolates from fatal cases of Brazilian spotted fever and comparison with *Rickettsia rickettsii* isolates from the American continents. J Clin Microbiol. 2014;52:3788–91. 10.1128/JCM.01914-14 25078908 PMC4187786

[8] Bermúdez SE, Castro AM, Trejos D, García GG, Gabster A, Miranda RJ, et al. Distribution of spotted fever group rickettsiae in hard ticks (Ixodida: Ixodidae) from Panamanian urban and rural environments (2007–2013). EcoHealth. 2016;13:274–84. 10.1007/s10393-016-1118-8 27068930

[9] Ammerman N, Beier-Sexton M, Azad A. Laboratory maintenance of *Rickettsia rickettsii*. Curr Protoc Microbiol. 2008;11:3A.5.1–21. 10.1002/9780471729259.mc03a05s11 PMC2725428 19016440

[10] Eremeeva ME, Dasch GA. Closing the gaps between genotype and phenotype in *Rickettsia rickettsii.* Ann N Y Acad Sci. 2009;1166:12–26. 10.1111/j.1749-6632.2009.04526.x 19538260

